# An Exploratory Data Analysis from Ovine and Bovine RNA-Seq Identifies Pathways and Key Genes Related to Cervical Dilatation

**DOI:** 10.3390/ani13132052

**Published:** 2023-06-21

**Authors:** Joedson Dantas Gonçalves, José Bento Sterman Ferraz, Flávio Vieira Meirelles, Ricardo Perecin Nociti, Maria Emilia Franco Oliveira

**Affiliations:** 1 Department of Pathology, Reproduction and One Health, School of Agricultural and Veterinarian Sciences, São Paulo State University, Via de Acesso Prof. Paulo Donato Castellane, s/n, Jaboticabal 14884-900, SP, Brazil; 2 Molecular Morphophysiology and Development Laboratory, Departament of Veterinary Medicine, Faculty of Food Engineering-FZEA, University of São Paulo, Av. Duque de Caxias Norte 255, Pirassununga 14635-900, SP, Brazil

**Keywords:** transcriptome, cervix, reproduction, sheep, cattle

## Abstract

**Simple Summary:**

Our results demonstrate differences and similarities in the mechanisms of cervical dilation in sheep and cows in the follicular and luteal phase. In cattle, 1961 genes were differentially expressed in the follicular phase and 1560 in the luteal phase. In sheep, 2126 genes were differentially expressed in the follicular phase and 2469 genes were more differentially expressed in the luteal phase. PI3K/Akt is a pathway that has been found in both species and seems to be an important pathway in the process of cervical relaxation. These results help us to better understand the mechanisms, especially in ewes where there is a difficulty in promoting cervical relaxation in the luteal phase to collect embryos.

**Abstract:**

The present study developed a review and exploration of data in public and already validated repositories. The main objective was to identify the pathways involved in ruminants’ cervical dilatation, which are conserved between cattle and sheep in the follicular and luteal phases of the reproductive cycle. In cattle, 1961 genes were more differentially expressed in the follicular phase and 1560 in the luteal phase. An amount of 24 genes were considered exclusively expressed from these. A total of 18 genes were in the follicular phase and 6 genes were in the luteal phase. In sheep, 2126 genes were more differentially expressed in the follicular phase and 2469 genes were more differentially expressed in the luteal phase. Hoxb genes were identified in both species and are correlated with the PI3K/Akt pathway. PI3K/Akt was also found in both cattle and sheep, appearing prominently in the follicular and luteal phases of both species. Our analyses have pointed out that the PI3K/Akt pathway and the Hoxb genes appear in prominence in modulating mechanisms that involve estrus alterations in the cervix. PI3K/Akt appears to be an important pathway in the cervical relaxation process.

## 1. Introduction

The anatomy of the ovine cervix is one of the main limiting factors for cervical transposition in sheep, due to the number, internal diameter, and distribution of cervical rings [1]. Even with cervical remodeling during the estrus phase, cervical penetration for artificial insemination procedures in ewes remains problematic [2,3]. The challenge is even greater during the luteal phase when embryo collection is performed [4]. A series of studies attempted to develop protocols with satisfactory cervical relaxation responses, for better application of reproductive biotechnologies [3]. The cervical dilatation mechanism in sheep is complex, involving different hormones (Prostaglandin H, Prostaglandin E2, oxytocin, estradiol, relaxin), glycosaminoglycans (hyaluronic acid, codroitin sulfate-4-6, dermatan sulfate, heparin sulfate and keratin sulfate), proteins (matrix metalloproteinases, glycoproteins, proteinases), cytokines (interleukins 1 and 8, tumor necrosis factor) and immune system cells (macrophages, leukocytes) [4]. Linked to these mechanisms, the anatomy of the ovine cervix has particularities compared to other species [1].

In ruminants, both at parturition and estrus, increased estrogen concentration appears to initiate a cascade of events that culminate in cervical relaxation [5]. There is a major obstacle to unraveling the mechanisms of cervical ripening, as the mechanisms in mammals are highly variable at parturition, which is the time of greatest dilatation. A practical example would be that in pregnancy and during childbirth, humans produce high concentrations of progesterone [6]. In mice, however, serum levels of progesterone are decreased at the end of pregnancy and during labor [7], as well as in sheep, rabbits, and mice [6].

The enzyme 5-α-steroid reductase (SRD5A1) is essential in cervical regulation and remodeling [8], being the expression of SRD5A1 mRNA necessary in the process of cervical ripening in mice, reported as a species-specific mechanism [7]. However, this enzyme is not found in guinea pigs, even though they belong to a rodent lineage with a basal branch [9]. It has also been reported that the enzyme 17β-hydroxysteroid dehydrogenase (HSD17B1), known mainly for its role in the synthesis of estradiol, is not expressed in the cervix of the opossum and rat, little expressed in the cervix of the armadillo, highly expressed in guinea pigs at all stages of the reproductive cycle, and in rabbits at the end of pregnancy [7]. Moreover, these are suggestive that in species with common ancestors, there are conserved cervical dilatation mechanisms, as well as variations of these mechanisms among species.

Furthermore, addressing those topics, review and data mining were carried out in public and already validated repositories. We hypothesized that differentially expressed genes and divergent biological processes would indicate differences and similarities related to cervical relaxation in cows and sheep. The main objective was to identify the pathways involved in cervical dilatation, which are conserved between cattle and sheep in the follicular and estrous phases of the reproductive cycle. Such results are important to better understand the mechanism of cervical dilatation in sheep and indicating signaling pathways, which may help to understand and improve the efficiency of cervical relaxation protocols. Thus, consequently, improving the application of reproductive biotechniques in ruminant species.

## 2. Materials and Methods

### 2.1. Bioinformatics for Obtaining and Processing Data

The bovine cervix transcriptomic data in the follicular and luteal phase were provided by Pluta et al. (2012) [10] and sheep by Abril-Parreño et al. (2021) [11], with the generated and/or analyzed datasets available in the NCBI Gene Expression Omnibus (https://www.ncbi.nlm.nih.gov/geo/) accessed on 25 November 2020, under accession number GSE38225 and GSE179486, respectively. Samples extracted from the study by Pluta et al. (2012) [10], were derived from 30 Limousin heifers with estrus synchronized by an intravaginal device (CIDR, Pfizer Animal Health) for 8 days. At 24 h before CIDR removal, they were injected intramuscularly with 2 mL of prostaglandin (0.25 mg/mL), (PGF2 Estrumate, Loughrea, Co., Galway, Ireland). The samples extracted from the study by Abril-Parreño et al. (2021) [11] were from the natural estrous cycle of about 40 multiparous ewes of the Belclare, Suffolk, NWS and Fur breeds.

As a criterion for data selection, only samples of studies that contained information related to the sequencing platform used were adopted, giving preference to studies that made them available in “raw” form, containing physiological information regarding the samples.

The files were downloaded in SRA format directly from GEO to the cluster of the Animal Improvement and Biotechnology Group at FZEA-USP. All data had the sequencing quality verified by the FASTQC software (v0.11.9), followed by the removal of reads according to the data quality (“trimming”) with the TRIM GALORE software (0.6.4_dev, 2019), both software from the Brabahan Institute, Cambridge, United Kingdom. (https://www.bioinformatics.babraham.ac.uk/projects/index.html, accessed on 25 November 2020).

After selecting the reads by quality, we checked the quality with the R fastqcr package (version 0.1.2, 2019) [12]. The samples that passed the quality test were then aligned with the reference genome of the species available at ENSEMBL (https://www.ensembl.org/info/data/ftp/index.html, accessed on 25 November 2020), software from the is based at the European Molecular Biology Laboratory’s European Bioinformatics Institute, Cambridge, United Kingdom. The genome version used for ovine was Oar_rambouillet_v1.0.104 and for bovine was used Bos_taurus.ARS-UCD1.2.104, with the version 2.10.2 of RSUBREAD software (Victoria, Australia) [13], using the software’s default parameters suitable for each type of sample library. Alignment quality was then verified and a final report was generated using MULTIQC software (version 1.10.dev0) (Seqera Labs, S.L. Barcelona, Spain) [14].

Software from the is based at the European Molecular Biology Laboratory’s European Bioinformatics Institute, Cambridge, United Kingdom.

### 2.2. Data Analysis, Identification of Gene Signatures, and Differences in Expression

The analysis of gene expression difference was performed with the version 1.36.0 of DESEQ2 package [15] of the R software [16] and exploratory data analysis was performed through principal component analysis (PCA) to the contrasts between the physiological situations of the cervix and between species.

For a gene to be considered differentially expressed, an adjusted *p*-value lower than 0.1 (padj < 0.1) was adopted by the Benjamini-Hochberg (“BH”) method, and an absolute value of “log2 fold change” greater than 0.6. For the representation of the gene expression values, the variance normalization transformation was used (function “varianceStabilizingTransformation” of the DESEQ2 package) which also served as an input for the data for the analysis of gene co-expression.

Gene co-expression analyses were used to search for transcriptional profiles, “gene signatures”, in samples at different physiological phases of the cervix and species. In this phase, we used the version 1.8.0 of CeTF package [17]. Moreover, if a gene had at least one count in all samples within the same group and zero counts in all samples from the other contrast group, the gene was considered exclusive in the first group.

After gene selection, gene ontology analysis and pathway enrichment were performed with the version 4.4.3 of ClusterProfiler package [18] of the R software. At first, we investigated the biological process and molecular functions and the cellular components involved. In addition, we investigated the cured pathways in KEGG (“Kyoto Encyclopedia of Genes and Genomes”) and REACTOME. To visualize the results, networks of gene interaction and pathways were built using ClusterProfile.

## 3. Results

### 3.1. Genes More Expressed in the Follicular and Luteal Phases of Cattle and Sheep

In cattle, 20,965 genes were considered expressed, while in sheep, 19,581 genes were considered expressed, and from those 15,082 genes were homologs genes expressed in both species. Furthermore, in bovine, a total of 1961 genes were found to be more expressed (padj < 0.1 and |log2foldchange| > 0.6) in the follicular phase and 1560 in the luteal phase. A total of 24 genes were considered exclusive of these 18 genes in the follicular phase and 6 genes in the luteal phase, while in sheep, a total of 4595 genes were considered differentially expressed (padj < 0.1 e |log2foldchange| > 0.6), with 2126 genes more expressed in the follicular phase and 2469 genes more expressed in the luteal phase. In addition, four genes were considered exclusive in the follicular phase.

The signaling pathways found have different functions and expression intensities according to the phase of the estrous cycle. Signaling pathways are linked to genes of different categories: adrenergic (calmodulin Like 5 (CALML5), leucine rich repeat containing G protein-coupled receptor 6 (LGR6), dopaminergic (protein phosphatase 1 regulatory inhibitor subunit 1B (PPP1R1B)), purinergic receptors (purinergic receptor P2Y2 (P2RY2)), growth factors (heparin binding growth factor (HDGF)), tumorigenesis (Lysine Demethylase 2A (KDM2A)), hematopoiesis (Myeloid Leukemia Factor 1 (MLF1)), chromatin regulation and condensation (Chromodomain Helicase DNA Binding Protein 4 (CHD4), transcriptional regulation (CCR4-NOT Transcription Complex Subunit 11 (CNOT11)), mucins (Mucin 1 (MUC1)), hormone ligands (ubiquitously expressed prefoldin like chaperone (UXT)), cartilagem (cartilage oligomeric matrix protein (COMP)) and genes involved in vasoconstriction, vasodilation, and muscle contraction (Histone Deacetylase 3 (HDAC3), Oxytocin Receptor (OXTR)).

**Figure 1:**
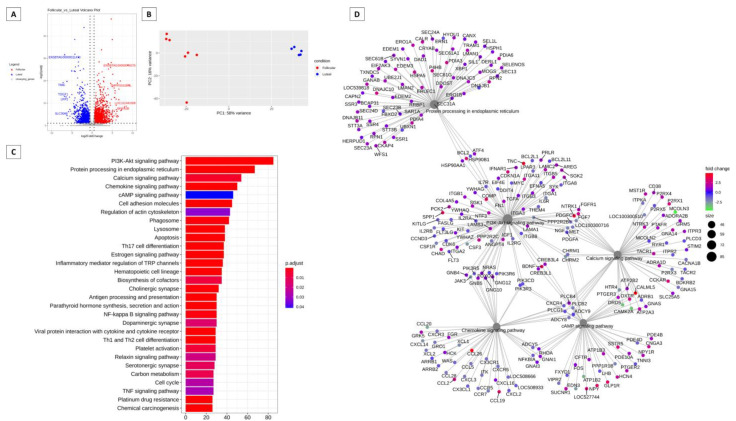
Summary of the different gene expression analysis in bovine cervix during the follicular and estrous phases. (**A**) Volcano plot comparing follicular and estrous phases with “log2FoldChange” being represented on the x axis and log2 of adjusted *p*-values on the y axis. (**B**) PCA analysis summarizing the cumulative variation in principal components 1 (PC1) and principal components2 (PC2) using only significant genes. (**C**) Barplot KEGG enrichment pathways analysis of differentially expressed genes, on x axis represents the number of genes, y axis the significant enriched kegg pathways and bar colors represents adjusted values with blue being the highest and red the lowest adjusted *p*-values, (**D**) Network analysis based on the pathways and differentially expressed genes during the follicular and luteal phase of the bovine cervix. Pathways circle size represents the number of genes in within enriched pathway and color represents the “log2FoldChange” values. Green are the lowest and red are the highest values.

Figure 1 presents a summary of the analysis of differences in gene expression in cattle in the follicular and luteal phases. The differentially expressed genes can be visualized in a volcano plot in Figure 1A, with the genes in red in the follicular phase and the genes in blue in the luteal phase. In the follicular phase, ENSBTAG00000048276 (trefoil factor 1), TMPRSS11B N-terminal-like (TMPRSS11BNL), LOC112441508 and BPI fold containing family A, member 2B (BPIFA2B) were differentially expressed. In the luteal phase, the most differentially expressed genes were ENSBTAG00000011470, transmembrane inner ear (TMIE), teratocarcinoma-derived growth factor 1 (TDGF1), LDL receptor related protein 2 (LRP2), and solute carrier family 30 member 8 (SLC30A8). In Figure 1B, we have the results of the PCA analysis, demonstrating the clustering of data. Figure 1C shows 30 different pathways that are modulated by these genes, with the PI3K/Akt pathway having the highest number of genes expressed. In Figure 1D, we have the network of genes with different expressions in which the interpellation of genes with the other pathways can be observed. We can observe the PI3K/Akt pathway in the center of the network.

**Figure 2:**
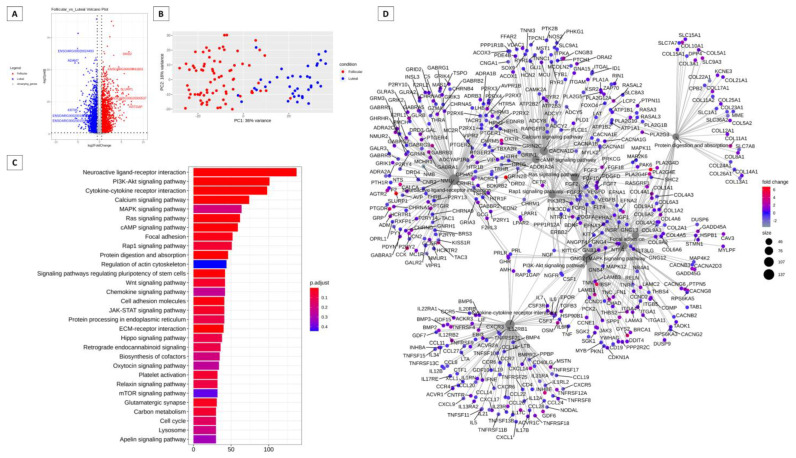
Summary of the analysis of the difference in gene expression of the ovine cervix in the follicular versus luteal phase. (**A**) Volcano plot of RNA sequencing (RNAseq) comparing follicular and estrous phases with “log2FoldChange” being represented on the x axis and log2 of adjusted *p*-values on the y axis. (**B**) PCA analysis summarizing the cumulative variation in principal components 1 (PC1) and principal components2 (PC2) using only significant genes. (**C**) Barplot KEGG enrichment pathways analysis of differentially expressed genes. The x axis represents the number of genes, y axis the significant enriched kegg pathways, and bar colors represents adjusted values with blue being the highest and red the lowest adjusted *p*-values, (**D**) Network analysis based on the pathways and differentially expressed genes during the follicular and luteal phase of the ovine cervix. Pathways circle size represents the number of genes in within enriched pathway and color represents the “log2FoldChange” values. Green are the lowest and red are the highest values.

Figure 2 presents the analysis of differences in gene expression in sheep in the follicular and luteal phases. The differentially expressed genes can be visualized in the volcano plot in Figure 2A, with the genes in red in the follicular phase and the genes in blue in the luteal phase. In the follicular phase, dopamine receptor D2 (DRD2), ENSOARG00020011601, secreted LY6/PLAUR domain containing 1 (SLURP1), ENSOARG00020000537, and keratinocyte differentiation-associated protein (KRTDAP) were differentially expressed. In the luteal phase, the differentially expressed genes were ENSOARG00020024493, ADAM metallopeptidase domain 7 (ADAM7), keratin, type II microfibrillar, component 5-like (KRT85), ENSOARG00020010220, and ENSOARG00020015599. In Figure 2B, we have the results of the PCA analysis, demonstrating the clustering of data. Figure 2C shows 30 different pathways that are modulated by these genes, with the neuroactive ligand-receptor interaction pathway having the highest number of genes expressed, followed by the PI3K/Akt pathway. In Figure 1D, we have the network of genes with different expressions in which the interrelationship of genes with the other pathways can be observed. We can also observe the PI3K/Akt pathway near the center of the network and peripherally the signaling pathways of Ras and RAP1.

### 3.2. Pathways That Can Be Modulated by Key Genes

**Figure 3:**
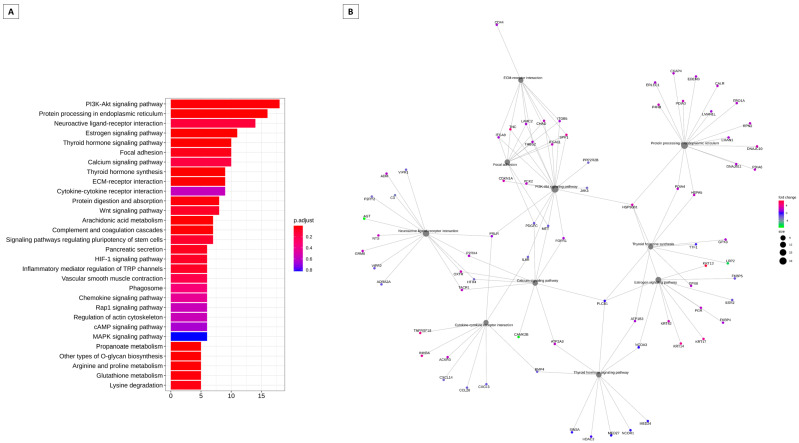
Analysis of enrichment of KEGG pathways that can be regulated by key transcription factors in cattle. (**A**) Barplot KEGG enrichment pathways analysis of values. Main gene pathways with interaction with key genes, on x axis represents the number of genes, y axis the significant enriched kegg pathways, and bar colors represents adjusted values with blue being the highest and red the lowest adjusted *p*-. (**B**) Network analysis based on the pathways and gene networks with interaction with key genes during the follicular and luteal phase of the bovine cervix. Pathways circle size represent the number of genes in within enriched pathway and color represents the “log2FoldChange” values. Green are the lowest and red are the highest values.

**Figure 4:**
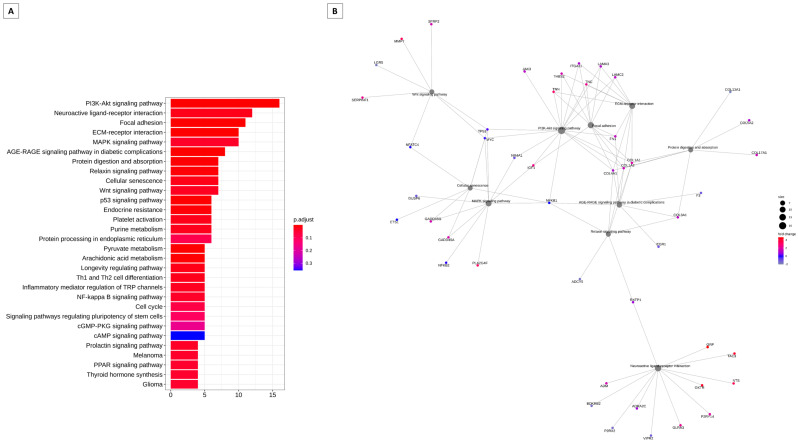
Analysis of enrichment of KEGG pathways that can be regulated by key transcription factors in sheep. (**A**) Barplot KEGG enrichment pathways analysis of values. Main gene pathways with interaction with key genes, on x axis represents the number of genes, y axis the significant enriched kegg pathways, and bar colors represents adjusted values with blue being the highest and red the lowest adjusted *p*-values. (**B**) Network analysis based on the pathways and gene networks with interaction with key genes during the follicular and luteal phase of the ovine cervix. Pathways circle size represents the number of genes in within enriched pathway and color represents the “log2FoldChange” values. Green are the lowest and red are the highest values.

Figure 3 summarizes the modulation of key genes and their possible targets in the bovine cervix (Figure 3A). These pathways may be modulated by key genes. The key gene is considered the highest degree of connectivity to other genes to promote a molecular event [19]. In the interaction network Figure 3B, again the PI3K/Akt pathway appears to be modulated. In Figure 4, we have the pathways related to the key genes in sheep. In Figure 4A, we have the main pathways related to key genes and the network of pathways with PI3K/Akt in the center, also modulated by these key genes (Figure 4B).

### 3.3. Pathways and Genes Can Be Modeled by Hox Genes 

**Figure 5:**
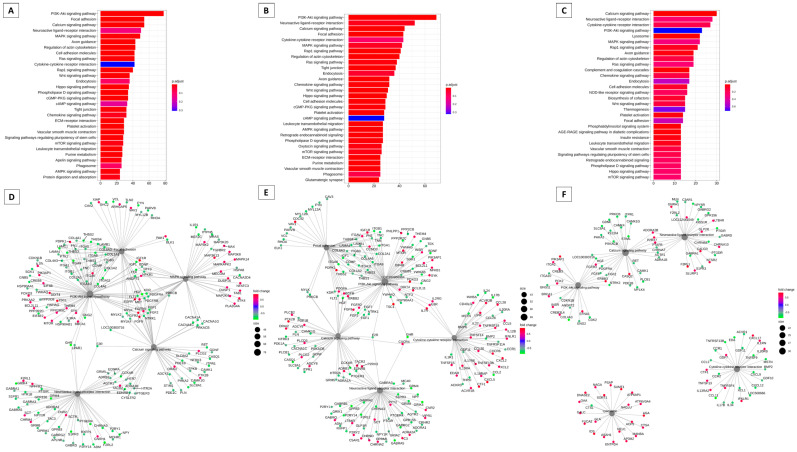
Analysis of enrichment of KEGG pathways that can be regulated by Hox genes in cattle. (**A**) Barplot KEGG enrichment pathways analysis of values. The main gene pathways with interaction with Hoxb3 gene, on x axis represents the number of genes, y axis the significant enriched kegg pathways, and bar colors represents adjusted values with blue being the highest and red the lowest adjusted *p*-values, (**B**) Barplot KEGG enrichment pathways analysis of values. The main gene pathways with interaction with Hoxb8 gene, on x axis represents the number of genes, y axis the significant enriched kegg pathways, and bar colors represents adjusted values with blue being the highest and red the lowest adjusted *p*-values, (**C**) Barplot KEGG enrichment pathways analysis of values. Main gene pathways with interaction with Hoxb9 gene, on x axis represents the number of genes, y axis the significant enriched kegg pathways and bar colors represents adjusted values with blue being the highest and red the lowest adjusted *p*-values, (**D**) Network analysis based on the pathways and gene networks with interaction with Hoxb3 gene during the follicular and luteal phase of the ovine cervix. Pathways circle size represents the number of genes in within enriched pathway and color represents the “log2FoldChange” values. Green are the lowest and red are the highest values, (**E**) Network analysis based on the pathways and gene networks with interaction with Hoxb8 gene during the follicular and luteal phase of the ovine cervix. Pathways circle size represents the number of genes in within enriched pathway and color represents the “log2FoldChange” values. Green are the lowest and red are the highest values, (**F**) Network analysis based on the pathways and gene networks with interaction with Hoxb9 gene during the follicular and luteal phase of the ovine cervix. Pathways circle size represents the number of genes in within enriched pathway and color represents the “log2FoldChange” values. Green are the lowest and red are the highest values.

**Figure 6:**
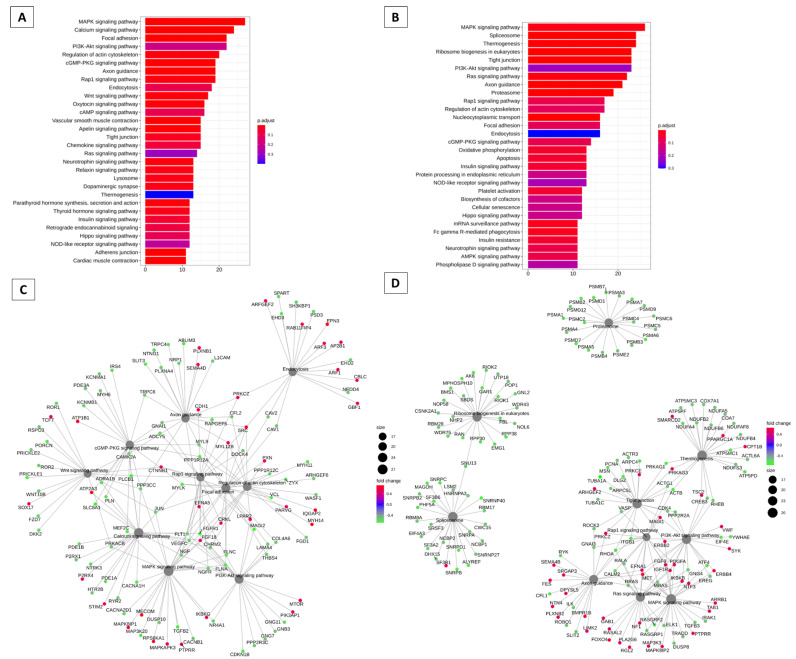
Analysis of enrichment of KEGG pathways that can be regulated by Hox genes in sheep. (**A**) Hoxb2, (**B**) Hoxb3, (**C**) Hoxb2, (**D**) Hoxb3. (**A**) Barplot KEGG enrichment pathways analysis of values. The main gene pathways with interaction with Hoxb2gene, on x axis represents the number of genes, y axis the significant enriched kegg pathways, and bar colors represents adjusted values with blue being the highest and red the lowest adjusted *p*-values, (**B**) Barplot KEGG enrichment pathways analysis of values. Main gene pathways with interaction with Hoxb3 gene, on x axis represents the number of genes, y axis the significant enriched kegg pathways, and bar colors represents adjusted values with blue being the highest and red the lowest adjusted *p*-values, (**C**) Network analysis based on the pathways and gene networks with interaction with Hoxb2gene during the follicular and luteal phase of the ovine cervix. Pathways circle size represents the number of genes in within enriched pathway and color represents the “log2FoldChange” values. Green are the lowest and red are the highest values, (**D**) Network analysis based on the pathways and gene networks with interaction with Hoxb3 gene during the follicular and luteal phase of the ovine cervix. Pathways circle size represents the number of genes in within enriched pathway and color represents the “log2FoldChange” values. Green are the lowest and red are the highest values.

In Figure 5, pathways and genes can be modulated by Hoxb genes in cattle (Figure 5) and sheep (Figure 6). Hox genes are essential in directing the further development of tissues in the embryonic stage [20]. It can be observed that the PI3K/Akt pathway appears to be modulated by these Hox genes, both in the follicular and luteal phases, appearing prominently in the interaction networks. It is also observed in the interaction networks, that several other genes may also be modulated by the Hoxb genes. These results were obtained using Spearman’s correlation with a *p* value < 0.05 and an absolute R-value greater than 0.5.

### 3.4. Hub Genes, 100 Most Expressed Genes and Genes Unique to the Follicular and Luteal Phase of Cattle and Sheep

In the supplementary material, the hub genes differentially expressed in cattle (Table S1) and sheep (Table S2) are demonstrated in the follicular (Positive Log Fold Change) and luteal (Negative Log Fold Change) phases. As a hub gene differentially expressed in the follicular phase in cattle, mucin 1 (MUC1) (Log Fold Change 1.57) has a fundamental role in the cervical mechanism. For sheep, the hub genes differentially expressed in the cervix during the follicular and luteal phases are shown in Table S2. The hub gene differentially expressed in the follicular phase of sheep was estrogen receptor 1 (ESR1) (Log Fold Change of 1.01). Estrogen receptor 1 (ESR2) was highly expressed in the luteal phase (Log Fold Change of −1.10).

Table S3 shows the 100 genes differentially expressed in the follicular and luteal phase of cattle found in the evaluation. The BPI fold containing family A, member 2ª (BPIFA2A) had the highest difference in expression (Log2 Fold Change of 10.49), being differentially expressed in the follicular phase. Differentially expressed in the luteal phase in cattle (Table S3), the angiotensinogen (AGT) (Log2 Fold Change of −7.72), is a participant in the renin-angiotensin system (RAS). The 100 differentially expressed genes in the follicular and luteal phase of the sheep cervix are shown in Table S4. The biggest difference in expression found was the trefoil factor 1 gene (TFF1 -Log Fold Change 5.31), being differentially expressed in the follicular phase, while in the luteal phase, ADAM metallopeptity domain 7 (ADAM7) was the most different expressed gene (Log Fold Change 5.58).

The genes considered exclusive in the bovine cervix from the follicular and luteal phases are presented in Table S5. An amount of 23 genes were considered exclusives. Of these, 17 genes were exclusive to the follicular phase (Positive Log Fold Change) and 6 genes were exclusive to the luteal phase (Negative Log Fold Change). BPI (BPIFA2B) was differentially expressed in the follicular phase of the bovine cervix (Log2 Fold Change of 8.01) and retinol dehydrogenase 16 (RDH16) (Log2 Fold Change of −5.30) was differentially expressed in the luteal phase.

## 4. Discussion

The cervix is a complex fibrous structure that undergoes structural changes according to the estrous cycle phase. In the follicular phase, the cervix is more open for the reception and transport of sperm [1]. In the luteal phase, the cervical canal is completely closed to protect against pathogenic microorganisms [21]. There is a biotechnical interest in cervical dilatation during the luteal phase in sheep due to the difficulty of transposition of the cervix. There is, however, more information related to cattle in the literature. Thus, a comparison of the mechanisms between sheep and cattle may be an alternative for a better understanding of the cervical relaxation event. Our findings point to molecular differences in the cervical physiology of cattle and sheep. Our results compare transcriptomic data from the cervix of cattle and sheep. However, the cattle data were from heifers and the sheep data were from multiparous females. Structurally and physiologically, there are differences in the cervix between heifers and multiparous. In heifers, the complete process of cervical maturation may not have occurred because they never had offspring. However, even with these implications, the heifers were submitted to a synchronization protocol, with the development of a follicular and luteal phase.

In the bovine follicular phase, four genes were found differentially expressed: TFF1, TMPRSS11BNL, LOC112441508, and BPIFA2B. TFF1 is encoded by an estrogen-responsive gene [22,23]. There are reports that the synthesis of secretory mucins is typically accompanied by the co-secretion of TTF peptides [24]. TFF peptides help maintain the surface integrity of mucosal epithelia [25]. TFF1 expression has been reported in small amounts in the human endocervix [26]. These triphilic peptides are involved in the protection and restoration of epithelia [26], in addition to binding to mucins [27,28] and mucin-associated proteins [29]. In the follicular phase, there is a large amount of cervical mucus production. This cervical mucus helps in cervical dilation, due to the release of substances that bind to collagen and cause distension of the fibers [30]. TMPRSS11B, also found in the follicular phase, is a member of the type II trypsin-like serine protease [31], expressed on the cell surface [32]. There are reports of data from the immunohistochemical analysis that show that TMPRSS11B is expressed in tissues of some types of cancer [32]. Serine proteases are related to the expression of pro-inflammatory cytokines [33]. The inflammatory process is important in the dilation mechanism, as it recruits defense cells. The neutrophil, for example, seems to be an important component for cervical softening, as the collagenase released by neutrophils is very important for the disruption of collagen fibers, which are the main structural elements of the cervix. This disruption causes distention of the organ [34].

Our findings show that BPIFA2B is differentially expressed in the follicular phase of the bovine cervix. Formerly known as bovine salivary protein 30b (BSP30B) [35] has reported its presence through immunohistochemical analysis in bovine salivary glands [36], being involved in the process of permeabilization of the plasmatic membrane of gram-negative bacteria [37]. In estrus, the cervix is open under the effect of estrogen, so the open channel allows the entry of bacteria from the normal flora of the vagina into the uterine lumen [1]. Thus, BPIFA2B may have its role related to antimicrobial activity. This gene is also considered exclusive in the bovine cervix in the estrous phase (Table S5).

In the luteal phase in cattle, the five differentially expressed genes were ENSBTAG00000011470, TMIE, TDGF1, LRP2, and SLC30A8. The TMIE already reported in adult mice and rats is expressed in various tissues [38]. However, the role of TMIE is still uncertain. There are only conclusions of alterations in mice with TMIE deficit in the inner ear, as this gene is involved in sensory mechanotransduction in cochlear hair cells [39]. In other tissues, whether in humans, rats, or other mammals, there are no reports of the functions of TMIE. STAT3 protein inhibitor has already been identified as a potential binding partner [40]. STAT3 is already reported in the remodeling of stroma and uterine epithelium in the luteal phase, mainly during embryonic implantation [41]. Thus, the TMIE may be involved in the mechanotransduction of signals involving structural changes in the cervix.

TDGF1 is a member of the epidermal growth factor (EGF)—Crypto-1/fibroblast growth factor (FRL1)-related ligand and acts as a ligand for activation of the src-Akt pathway [42] and is involved in embryogenesis and tumorigenesis [43,44,45]. Furthermore, TDGF1 may be involved in epiblastic specification of bovine blastocysts [46]. Secreted soluble forms of TDGF1 may also activate the PI3K/Akt pathway [47]. In addition, it may have an essential role in the regulatory processes of stem cell proliferation and differentiation [48]. Furthermore, this gene is related to cell stimulation, growth, and differentiation, and its action may be linked to cervix physical changes during the estrous cycle. Another gene differentially expressed in the luteal phase in cattle is LEP2, an endocytic receptor strongly expressed in steroid-responsive tissue and epithelial cell types, already reported in male and female reproductive organs [49]. It may also have effects on cervical mechanical characteristics because its action on female reproductive systems is related to the alteration of uterine architecture, being positively regulated by progesterone (P4).

In the ovine follicular phase, five genes were differentially expressed: DRD2, ENSOARG00020011601, SLURP1, ENSOARG00020000537, and KRTDAP. DRD2 is considered a dopamine receptor with a role in motor control and neuroendocrine activities [50]. In male reproductive organs, DRD2 has been linked to the stimulation of penile erection, mediated by the oxytocin pathway [51]. In females, oxytocin is responsible for the excitability and contractility of the uterus [52]. In this way, DRD2 as a dopaminergic receptor can act directly or indirectly for these stimuli to occur. SLURP1 facilitates the functional development of T cells and suppresses the production of TNF-a (Tumor Necrosis Factor-alpha) by T cells, secretion of IL-1 b and IL-6 by macrophages, and in humans, the secretion of IL-8 by the intestine [53]. This affinity for interleukins and tumor necrosis factor may be linked to the mechanism of cervical dilatation, as these interleukins actively act on the cellular remodeling of the cervix and, consequently, on cervical relaxation [54,55]. Oxytocin has an important effect on dilation, in which there is an increase in oxytocin receptors in the cervix, due to high concentrations of follicular estradiol [56]. The binding of oxytocin to its receptors promotes the production of Prostaglandin E2, the latter promoting cervical dilation through its action on smooth muscle cells and joint tissue [57]. These 2 genes are also highlighted among the 100 genes most differentially expressed in the follicular phase of sheep (Table S4).

In the luteal phase, the most differentially expressed genes were ENSOARG00020024493, ADAM7, KRT85, ENSOARG00020010220, and ENSOARG00020015599. More recently, it was reported that ADAM7 overexpression strongly promoted cell proliferation, migration, invasion, and inhibited cell apoptosis in trophoblastic cells [58]. In addition, there are reports that matrix metalloproteinases, such as ADAM7, promote collagen degradation, to regulate the survival, growth, migration, and invasion of cancer cells [59,60]. As collagen is one of the components of the cervix, it is suggested that this gene also acts in this organ, causing modifications. During cervical dilatation, collagen bundles are separated by increased water perfusion in the cervical extracellular membrane and collagen degradation by matrix metalloproteinases [61]. The findings by Kershaw et al. (2007) and Rodríguez-Piñón et al. (2015) [62,63] show a higher proportion of collagen in the luteal phase compared to the estrus phase. This gene is also highlighted among the 100 genes most differentially expressed in the luteal phase of sheep (Table S4).

In our analysis, Hoxb genes were found in both cattle and sheep and their regulation may be involved in the PI3K/Akt pathway. Hox genes are considered members of the homeotic transcription factor family. They are the ones that will specify and direct the formation of tissues in the embryo [20]. Hox genes are involved in the gearing of proteins regulating transcription factors responsible for the development of the axial system in vertebrates [64,65,66]. Hoxb2 appears to be specifically involved in motor neuron development [67]. Hoxb9 is reported in the mechanism of mammary gland development [68], regulation of components of the chick respiratory tract [69], and related to the regulation of carcinoma and adenocarcinoma in humans [70,71]. Hoxb8 is related to hematopoietic cells and may even generate myeloid cells capable of self-renewal [72]. In addition, its expression is reported since embryonic development [73]. De las Heras-Saldana et al. (2019) [74] provided evidence that the differential expression of some Hox genes, such as Hoxb2, Hoxb4, and Hoxb9, may be involved in muscle differentiation, where these genes seem to modulate the muscle fate of satellite cells during myogenesis. However, overall, the role of these Hox proteins in the cervical epithelium is still unclear [75]. These Hoxb genes appear to be involved in several biological processes, such as the formation of organs and muscles [76]. When the cervix undergoes structural, chemical, and biological differences throughout the estrous cycle [1], these Hox genes can modulate some mechanisms of cervical tissue transformation.

The PI3K/Akt signaling pathway was found in both cattle and sheep and could be regulated differently in both species (Figure 1 and Figure 2). PI3K/Akt is involved in different signaling processes, ranging from apoptosis to cellular metabolism processes [77]. The PI3K/Akt pathway appears prominently in both species and may be involved in signal transduction mechanisms in different systems and organs. For communication between cells to occur, signaling pathways are necessary to command and mediate various functions, whether migration, differentiation, proliferation, or death [77]. PI3K/Akt plays important roles in control and balance in multicellular organisms [77]. Some studies have already documented the PI3K/Akt pathway signaling cascade and its involvement in the regulation of cell survival and apoptotic inhibition [78], as well as acting in the uptake of glucose and consequent involvement in the metabolism [79,80]. The PI3K/Akt pathway has been reported as an important survival pathway of eukaryotic cells, with the Akt serine/threonine kinase considered the key signaling point [81]. Furthermore, activated Akt directs an anti-apoptotic signal, thus protecting even cancer cells [82]. Its involvement is also related to the regulation of the functional activity of several proteins which trigger different biological responses [83,84,85,86]. Thus, phosphorylation or dephosphorylation can control or regulate specific biological processes, catalyzing enzymes, regulating ion channels, transcription factors, intracellular protein localization, cytoskeleton regulation, and receptor activity [87]. The signaling pathway of the neuroactive ligand-receptor interaction has neural function through intracellular receptors and may also regulate gene expressions [88]. Even though it is the most expressed pathway in sheep, some genes are in common connection with the PI3K/Akt signaling pathway (Figure 2). The interaction of the neuroactive ligand-receptor interaction pathway has already been reported with the extracellular matrix (ECM) in humans, having some genes in common [89].

Some genes found in our study have already been reported to be involved in the PI3K/Akt pathway (TDGF1, Hoxb2, DRD2) [47,67,90]. However, we showed that PI3K/Akt may be modulating most of the genes presented, as it appears in the analysis as one of the most expressed pathways in both sheep and cattle. The Hoxb genes are also modulating or are modulated by the PI3K/Akt pathway in both species (Figure 5 and Figure 6). As this pathway is related to several biological processes, it may be an interesting pathway in the regulation of cervical relaxation mechanisms. Its stimulation or blockage can trigger cervical changes, as there is a relationship with several genes that supposedly participate in the process of dilation of the cervix.

The key gene most differentially expressed in the follicular phase in cattle was MUC1 (Table S1). This gene has a fundamental role in the cervical mechanism, as there is an interaction between cervical mucus and collagen cells, as this mucus contains specific glycoproteins and enzymes that can act directly or indirectly on collagen [30]. In addition, this gene is related to the formation of mucous barriers due to its presence on the surface of ciliated and secretory cells [91]. Cervical mucus can vary in its properties according to the estrous phase, attributing to the physiological need for sperm transport, acting as an antimicrobial agent, and also being a natural barrier in the luteal phase of the cycle and during pregnancy [92]. Mucin genes are already reported to show a strong correlation with increased estradiol levels [10] and that high cervical MUC1 expression during estrogen dominance enhances its known role as an antimicrobial barrier [93,94] especially when the cervix is open.

The hub gene most differentially expressed in the follicular phase of sheep was ESR1, while in the luteal phase, it was ESR2 (Table S2). The presence of ESR1 is the main determinant of the regulation of the OXTR gene in the endometrial epithelium [95]. These are involved in muscle relaxation and contraction [56]. ESR2 has been reported in all uterine and uteroplacental tissue compartments, with constant expression throughout early pregnancy [96]. These receptors play a fundamental role in dilatation, as estradiol is fully involved in the cervical relaxation mechanism, as it indirectly induces smooth muscle relaxation and extracellular matrix remodeling [61]. In addition, the natural cervical relaxation in estrus is due to high serum concentrations of estrogens, which allow the cervical opening for the passage of spermatozoa [1].

Differentially expressed in the follicular phase in cattle (Table S3), BPIFA2A is characterized as a protein that is housed in leukocytes and has antibacterial effects, specifically on gram-negative bacteria [97,98]. This protein is considered bactericidal [99], and its role may be related to antimicrobial activity in the cervical canal. Found in the luteal phase, AGT (Table S3), is related to the maintenance and regulation of the renin-angiotensin system (RAS) and, consequently, to maintain balance in the regulation of blood pressure [100]. Its role seems to be involved in the vasoconstrictor effect, apoptosis, angiogenesis, and cell proliferation in several cell types [101]. In sheep, AGT encodes an angiotensin II product, described as a vasoconstrictor and regulator of fetoplacental angiogenesis in the placenta [102]. In the cervix, its role has not yet been elucidated. However, it is already reported that there is an effect of angiotensin on the production of estradiol, but this mechanism is still not fully understood [103,104]. According to Giani et al. (2007) and Sampaio et al. (2007) [105,106], angiotensin may play a role in estradiol production through PI3K/Akt signaling.

## 5. Conclusions

Our analyses indicate that the PI3K/Akt pathway and the Hoxb genes can modulate and be modulated in mechanisms involving cervical changes in cattle and sheep. In vivo studies of blockade or stimulation of this pathway should be performed to assess cervical relaxation in ewes, especially in the luteal phase, in which embryo collections are performed via the transcervical route.

## Data Availability

The data that support the findings of this study are available from the corresponding author upon reasonable request.

## Supplementary Materials

The following supporting information can be downloaded at: https://www.mdpi.com/article/10.3390/ani13132052/s1, Table S1: Hub genes in the bovine cervix in the follicular and luteal phases; Table S2: Hub genes in the sheep cervix in the follicular and luteal phases; Table S3: The 100 most expressed genes in the follicular and luteal phase of the bovine cervix; Table S4: The 100 most expressed genes in the follicular and luteal phase of the sheep cervix; Table S5: Genes exclusive to bovine cervix in the follicular and luteal phase.
