# Supplementary material for: An Exploratory Data Analysis from Ovine and Bovine RNA-Seq Identifies Pathways and Key Genes Related to Cervical Dilatation
Source: Animals (Basel). 2023 Jun 21;13(13):2052. doi: 10.3390/ani13132052 (PMC10339884; doi:10.3390/ani13132052)
Supplement: Supplementary file 1 [file animals-13-02052-s001.zip › animals-2236994-supplementary.pdf]

Supplementary Materials

Table S1: Hub genes in the bovine cervix in the follicular and luteal phases.

Gene	ID	**Log Fold Change**	**Dif Means**	gini_1	gini_2	Mean1	Mean2
ENSBTAG00000046878	ENSBTAG00000046878	0.323090905795746	0.0974687086131434	0.235706990262491	0.150256817634492	2.12500164212631	2.02753293351317
HMG20A	ENSBTAG00000020441	-0.097811620375919	-35.6041149551268	0.150758398078968	0.055365825614059	223.899762583899	259.503877539026
ZNF419	ENSBTAG00000017613	-0.554027692420169	-4.47580907197521	0.194980575222539	0.0806871481349731	7.82256686356427	12.2983759355395
KDM2A	ENSBTAG00000044032	-0.278163633888021	-28.8356191176694	0.0676107738152934	0.0164675188247649	70.3885376362167	99.224156753886
MLF1	ENSBTAG00000004126	-0.325266641627905	-0.232716529183624	0.293391867988911	0.125574877185134	0.829843253362801	1.06255978254643
EAF1	ENSBTAG00000019202	0.153662877986998	-0.68872834124987	0.0755208628590565	0.0598917016902881	17.4318724379855	18.1206007792354
ATXN1L	ENSBTAG00000046255	-0.386820917363827	-58.7855538815191	0.129073736081567	0.0708260078213465	105.67588459104	164.461438472559
PLCB1	ENSBTAG00000008338	0.0263776314191702	0.25523379759435	0.3722674457735	0.225286511119357	11.4034538041402	11.1482200065458
MTA3	ENSBTAG00000004073	-0.737863953960057	-1.02136351434001	0.166499515235935	0.0333845024301732	1.30144418349839	2.3228076978384
CHD4	ENSBTAG00000014734	-0.130782859183929	-6.78320930715659	0.0832530342936934	0.0334759724568583	24.6016587547516	31.3848680619082
SERTAD3	ENSBTAG00000010502	0.137178762257812	-3.30498776424935	0.119601097591744	0.0620024387453041	41.5836528288874	44.8886405931367
PAXIP1	ENSBTAG00000017505	-0.402946136882788	-2.80396781033697	0.0995477836732697	0.0366688036738553	4.9062910235212	7.71025883385816
CNOT11	ENSBTAG00000008267	-0.241937023687223	-3.47670020759057	0.061492036316046	0.0804970145539508	8.41602899555025	11.8927292031408
HDAC3	ENSBTAG00000017360	0.224215468342189	-0.0142271687011091	0.0659028887210294	0.0560509519930072	4.01614752448022	4.03037469318133
PPP1R13L	ENSBTAG00000020689	-0.0700090234373506	-4.54311978602093	0.148848115499664	0.0762984316276214	17.9724270886137	22.5155468746347
MED24	ENSBTAG00000021468	-0.286137172499933	-11.3832112785057	0.174332595167555	0.0953427522916272	25.4681736958781	36.8513849743838
MAPK13	ENSBTAG00000010007	0.660204589458536	4.16958148725283	0.241961568190942	0.0805168388596126	15.887714391138	11.7181329038852
DNM2	ENSBTAG00000013362	-0.284789014273538	-56.6815812569633	0.117254565300117	0.0497941096933369	132.8287126338	189.510293890764
POLR2G	ENSBTAG00000009483	-0.187446185805971	-25.7066726820744	0.0910016855977341	0.0702135471573257	78.314586446408	104.021259128482
HDGF	ENSBTAG00000039793	0.320506261786834	5.48429561132043	0.182160554072257	0.0155602034016393	104.905183948395	99.4208883370741
NCOA7	ENSBTAG00000046503	0.15382574209718	0.173062698618675	0.271179627708595	0.0973093319484125	2.000303035604	1.82724033698532
POLR1A	ENSBTAG00000006481	-0.251278069173389	-2.41805053225654	0.154504542773181	0.0313159547267263	5.85852213794489	8.27657267020144
PCBD1	ENSBTAG00000011866	-1.00054718470556	-55.2553850397195	0.0998312648474659	0.162383011750733	40.7776929514488	96.0330779911683
FHL2	ENSBTAG00000001086	0.485372105894402	1.94472104627881	0.136606376657373	0.086493415258272	11.2951894937647	9.35046844748589
PARP1	ENSBTAG00000000837	-0.363783527108227	-15.4769191237788	0.0398020461504979	0.0849686302412875	32.177613854876	47.6545329786548

CTNND1	ENSBTAG00000002411	-0.248202512839939	-32.9118857998668	0.0413229709870337	0.0641898744322226	87.4259484940269	120.337834293894
HMGB2	ENSBTAG00000015101	0.0951912890051601	0.00753577861681576	0.259541951499165	0.0716464616677119	1.7657280372761	1.75819225865928
GTF2IRD1	ENSBTAG00000006212	0.151755402219555	0.0407206459938303	0.105740675175251	0.0323560133039303	6.29552455959678	6.25480391360295
NCOR1	ENSBTAG00000013271	-0.244658789238636	-27.0169447226311	0.021559098048567	0.0720353525779446	72.102492583073	99.1194373057041
PSMD9	ENSBTAG00000004179	-0.0370806282632892	-18.7471768141625	0.110027436113434	0.07130251572394	82.0294603913207	100.776637205483
AJUBA	ENSBTAG00000012724	-0.529155495055643	-1.61279835853387	0.208629847040172	0.0818242218433426	2.18200704736216	3.79480540589603
CBX8	ENSBTAG00000009107	-0.210447734296982	-1.46510266876569	0.172042185294599	0.079220029624588	3.70213479634887	5.16723746511456
UXT	ENSBTAG00000015820	0.827741369211141	0.548291658019708	0.0934388038067035	0.0479168121332011	1.54252077195433	0.994229113934617
DNMT3A	ENSBTAG00000021143	-0.434134133243991	-8.14509367130768	0.0567895353290105	0.0780697518952753	14.0972897830828	22.2423834543905
MUC1	ENSBTAG00000017104	1.5759491672159	42.8763687088901	0.223296975494568	0.121048657094685	69.9981017066941	27.1217329978039
CASZ1	ENSBTAG00000019818	-0.476760960310648	-13.6617530505828	0.179112085831366	0.0539784354556604	21.6177863357526	35.2795393863354
AKAP8L	ENSBTAG00000010439	-0.830693748870828	-15.8819429664438	0.0754461648341227	0.0822111796622625	14.4085390060616	30.2904819725054
TERF2IP	ENSBTAG00000015686	0.318015273128463	24.8849738754909	0.155105751275222	0.0616124817431729	192.18388228105	167.298908405559
ERBB2	ENSBTAG00000021798	-0.292809880067697	-10.6401816776785	0.175583223517799	0.0542535757904137	24.4191302538498	35.0593119315283
HOXB5	ENSBTAG00000045835	0.00864829046434397	-1.04952052333382	0.225358852349498	0.067074862889544	6.37975612024244	7.42927664357627
MRTFB	ENSBTAG00000008728	-0.624873241342757	-190.379788029047	0.0992043214792029	0.0742666803137045	265.581270076209	455.961058105256
PRR13	ENSBTAG00000026916	0.415231450983471	3.46838948028413	0.0976592569056149	0.0665391345487741	24.3860100653748	20.9176205850907
TAF5L	ENSBTAG00000034225	0.00685040867780777	-1.56754730946581	0.0698815481371179	0.0262408554417917	8.90813267844142	10.4756799879072
LBH	ENSBTAG00000026111	1.62876889195243	68.3472801677114	0.195756522893946	0.206335254191895	111.832884919684	43.4856047519724
KDM5B	ENSBTAG00000006175	-0.0384307613041076	-93.2508657006142	0.0526802723220755	0.0472437077893247	522.471897421563	615.722763122177
HIPK2	ENSBTAG00000017860	-0.517739699942934	-0.687623003232602	0.200709874829096	0.154832128115389	0.961722414598122	1.64934541783072
RBPMS	ENSBTAG00000033727	-0.0821872535967167	-3.3341276480752	0.181214002198717	0.0477743446953832	17.2679523423757	20.6020799904509
SUFU	ENSBTAG00000021068	-0.430880083902824	-48.333046361498	0.152733752122834	0.0565977788653143	82.2086505893704	130.541696950868
DDIT3	ENSBTAG00000031544	0.16505738512273	-0.0151195362049265	0.0897820366710559	0.0710138370339491	0.570553272993806	0.585672809198732
SIN3A	ENSBTAG00000009985	-0.330965972069626	-199.903782856967	0.0237860262980164	0.0423392114721217	438.465354755268	638.369137612235
TTF2	ENSBTAG00000015392	0.0335898096923506	-0.184857914906793	0.0783349867149877	0.0942793689211042	1.45788406468644	1.64274197959323
GLI3	ENSBTAG00000010671	-0.489594438274782	-1.10526185431398	0.112982120825763	0.206483303510563	2.16201057768916	3.26727243200314
WWTR1	ENSBTAG00000007814	0.336308362701791	2.32037189128911	0.15467211358249	0.130324703370644	15.9233777818065	13.6030058905174
MED27	ENSBTAG00000000382	-0.219793153551425	-1.2610014618665	0.0864156924314682	0.0700801254637184	3.29738598755132	4.55838744941782
NIF3L1	ENSBTAG00000018282	0.0452452934566299	-0.253769631446751	0.097878942781277	0.0746797978984681	1.97507604923806	2.22884568068481

ZMYND8	ENSBTAG00000013114	0.0407214186397831	-4.79767537392712	0.103737274353588	0.0880773620047245	37.4699268041671	42.2676021780943
AKAP8	ENSBTAG00000001807	-0.739349226986793	-27.0594230934803	0.0360170729294317	0.116894580925637	28.9289675219679	55.9883906154482
PIR	ENSBTAG00000009477	-0.769558551092231	-1.97221922120661	0.146186261809711	0.274238516276698	1.8700384879575	3.84225770916411
CBX5	ENSBTAG00000006246	-0.200690663869542	-1.26864230591765	0.234335433665849	0.275105125962369	3.13939720403573	4.40803950995339
CHCHD2	ENSBTAG00000040295	0.198797918675193	0.111341607413301	0.0273742790011501	0.0231861619172467	16.7999379301479	16.6885963227346
EYA3	ENSBTAG00000043989	-0.781036767689086	-4.17243304911453	0.108507282862601	0.188914797769056	4.23290512769502	8.40533817680956
ERCC3	ENSBTAG00000020777	-0.301128827259608	-2.23947701494021	0.0584551881090467	0.0616981470877299	5.37139029961986	7.61086731456008
TTF1	ENSBTAG00000018710	-0.640128183027747	-2.85342180007701	0.165523813941282	0.143836730900824	3.51149834479708	6.36492014487409
CTBP2	ENSBTAG00000003397	-0.302457620831959	-24.7988961520473	0.0257536836489903	0.118241347030093	56.7611236001937	81.560019752241
ELOA	ENSBTAG00000026585	-0.190045863234437	-1.17622664833641	0.073625795492223	0.127380982139692	3.43279096938085	4.60901761771726
NSD3	ENSBTAG00000001529	-0.40020208685063	-2.72480938395963	0.120820044907004	0.191095218161158	5.12458954288114	7.84939892684077
SETDB1	ENSBTAG00000000098	-0.137270406082548	-2.54274015267468	0.0630142898147256	0.0464996484970122	10.3896364685647	12.9323766212394
XRCC6	ENSBTAG00000006103	-0.0622779066596389	-3.09018777209705	0.0565803523281743	0.0696450581495576	15.0992560366452	18.1894438087423
EYA2	ENSBTAG00000013336	-0.526791657200815	-33.2314654534435	0.221314203650821	0.194058006897459	48.5464792331013	81.7779446865448
KMT2C	ENSBTAG00000024199	-0.454799322092206	-4.78000998816025	0.138748417396087	0.167401441851236	7.82611348466135	12.6061234728216
ADNP2	ENSBTAG00000005916	-0.116163759756263	-10.3260474111518	0.0413737094312209	0.0824022806520881	41.0894510663885	51.4154984775403
PHF8	ENSBTAG00000013289	-0.278043931329241	-0.560352132539939	0.0819503481526139	0.0691964908899573	1.37728642021192	1.93763855275186
CHD7	ENSBTAG00000021841	0.138016988592552	-1.11585011467081	0.0574049388056796	0.118062717809342	16.1083784498887	17.2242285645595
PRMT2	ENSBTAG00000005503	0.223498490763962	0.477582624873879	0.0661436292350077	0.165762403066505	10.9413196563231	10.4637370314492
ANP32A	ENSBTAG00000012365	-0.545482901740455	-12.360971099161	0.049446597918387	0.0820583915000928	18.4424967002574	30.8034677994184
TLE4	ENSBTAG00000003532	-0.372693879438917	-3.30437445747314	0.134462867061302	0.1435660025412	7.82102573885292	11.1254001963261
TAF9B	ENSBTAG00000000895	0.620916141230957	2.54431516417144	0.116160062199806	0.0617225979069945	9.38427087439089	6.83995571021944

Table S2: Hub genes in the sheep cervix in the follicular and luteal phases.

Gene	ID	**Log Fold Change**	**Dif Means**	gini_1	gini_2	Mean1	Mean2
HOXB3	ENSG00000120093	-0.0444430789760876	-0.314671750814491	0.151961424329116	0.0935109779778038	3.47504846716703	3.78972021798152
ZNF34	ENSG00000196378	-0.675857205978606	-8.6660830579716	0.162368238741957	0.127249655493742	12.6954084135128	21.3614914714844
ESRRA	ENSG00000173153	0.359070099652815	1.62194048171043	0.153013490022434	0.104512468758234	9.55126023190595	7.92931975019552
PREB	ENSG00000138073	0.0444018064928695	-54.2517992481799	0.114569552788885	0.0607799165187727	1456.45163360534	1510.70343285352
NFIL3	ENSG00000165030	-0.0169807492978161	-0.249525902434344	0.152315853464092	0.16227380802779	2.93097132679661	3.18049722923096
ERF	ENSG00000105722	-0.0257949519021511	-1.33169227757189	0.0732487953125763	0.0756843804643073	16.7055796454344	18.0372719230063
ZFP69	ENSG00000187815	-0.0163665173166672	-0.465628586900626	0.108476231114715	0.110361484286911	6.39583826781362	6.86146685471425
NFATC4	ENSG00000285485	-0.0613435394215662	-0.052688596466541	0.190533197525829	0.187300817469598	0.473455585261797	0.526144181728338
HOXB2	ENSG00000173917	0.12631164230233	0.0212668761551627	0.124500139406979	0.0872380906881615	0.858067660572667	0.836800784417504
MEIS3	ENSG00000105419	-0.193128262018687	-0.885761589655636	0.176077876350497	0.189835438101973	4.06396677084364	4.94972836049927
EHF	ENSG00000135373	0.0551965703476608	-1.67338329120975	0.196017368527114	0.215629831212362	66.8751223503233	68.548505641533
CIZ1	ENSG00000148337	-0.131516997169159	-14.9721076043833	0.0593695173976606	0.0521777490981299	93.0444150402619	108.016522644645
ZNF750	ENSG00000141579	0.0594469909348513	-0.0322659848142932	0.220586172762167	0.257569746526407	1.54308108925488	1.57534707406917
SPDEF	ENSG00000124664	0.0480488621999171	-0.159760552038249	0.279871408616987	0.296337915564375	4.20658317236293	4.36634372440118
SIX1	ENSG00000126778	-0.254342448908673	-0.941013987856023	0.199799343567225	0.175946570997883	3.51375109242425	4.45476508028027
MAF	ENSG00000178573	0.549186451698951	0.372364257677024	0.244690329596164	0.226006961680613	1.33099585461996	0.958631596942931
NFX1	ENSG00000086102	-0.548530206406819	-0.189164549938	0.0701254664301291	0.072639897843982	0.345328133745948	0.534492683683947
ZNF276	ENSG00000158805	-0.121635166801796	-2.87300000942937	0.0582528997995442	0.0606613116519825	18.5651606480843	21.4381606575137
SMARCE1	ENSG00000073584	0.196547198465193	0.193985480805279	0.0589504960384857	0.0466811287881742	2.67820622279822	2.48422074199294
ZNF652	ENSG00000198740	-0.155346848428834	-3.85356440022137	0.12897091404807	0.18332601432775	21.0685773436668	24.9221417438882
GLIS3	ENSG00000107249	-0.749938080238254	-0.0820176207717817	0.160327753176676	0.234603031391278	0.104947898379246	0.186965519151028
ZNF710	ENSG00000140548	-0.275250488787159	-0.449058234688207	0.120555352842691	0.0637561280594882	1.59255599435058	2.04161422903879
NFKB2	ENSG00000077150	0.0739894498970104	-0.0609063251642361	0.154318775631769	0.16045039234085	13.8668550230431	13.9277613482074
MYC	ENSG00000136997	-0.220300417128693	-207.15784306916	0.135040985351753	0.130502425908539	866.605574159048	1073.76341722821
ESR1	ENSG00000091831	1.01079315236001	60.3619813538215	0.173406092548588	0.188435638047441	128.045272456939	67.6832911031179
PRDM2	ENSG00000116731	-0.442546886518727	-40.3011041728139	0.115748330406608	0.228262116953901	91.2567397626774	131.557843935491
STAT6	ENSG00000166888	0.00992095296381899	-0.780117575573508	0.0835705175230118	0.0855312731787687	15.5401156278796	16.3202332034531
PKNOX2	ENSG00000165495	0.340110364637196	0.537233345695989	0.186571725957367	0.269787541268671	3.35377204074548	2.81653869504949

ESR2	ENSG00000140009	-1.10402057614656	-4.06063822919675	0.449806144012118	0.49348544621593	3.19638564515389	7.25702387435064
NFKB1	ENSG00000109320	0.0149791993426645	-44.1303882919558	0.110342446846259	0.0973525054412114	1005.12136278387	1049.25175107582
TP53	ENSG00000141510	0.199871045564648	0.350397278968463	0.107219555382859	0.108659714239451	4.66407760591478	4.31368032694632
MEF2D	ENSG00000116604	0.209997237112109	0.292505529228725	0.0710573153580517	0.0850085171579094	3.46446678143662	3.1719612522079
ETS1	ENSG00000134954	0.018217625848141	-0.0500412714152569	0.138495376636028	0.181634901701882	1.01989553583063	1.06993680724589

Table S3: The 100 most differently expressed genes in the follicular and luteal phase of the bovine cervix.

Gene	ID	**Log Fold Change**	**Dif Means**	gini_1	gini_2	Mean1	Mean2
BPIFA2A	ENSBTAG00000009144	10.4876703575293	1.33314666767432	0.536551689835304	0.8	1.33379545013532	0.000648782460996904
TMPRSS11BNL	ENSBTAG00000048377	9.13963845167615	44.6748103458901	0.492638564074233	0.416231380534227	44.7637138270306	0.0889034811405015
ENSBTAG00000048276	ENSBTAG00000048276	8.83451158367456	11.0798911087455	0.260716716149161	0.368647838134362	11.1076696230359	0.0277785142904404
LOC112441508	ENSBTAG00000031375	8.43389967371738	1.48923886546534	0.528562019526543	0.623714018536848	1.49442237481876	0.00518350935341578
BPIFA2B	ENSBTAG00000019752	8.01169488659356	0.627058853372568	0.495585367076419	NA	0.627058853372568	0
AGT	ENSBTAG00000012393	-7.72323486386274	-2.83968274534543	0.391639105233969	0.769482777513028	0.0107830276558119	2.85046577300124
TMPRSS11D	ENSBTAG00000001925	7.2661091230698	5.64468362774846	0.535834114542948	0.401650565146232	5.68581144313726	0.0411278153887952
CAMK2B	ENSBTAG00000012653	-7.19841629076593	-36.6400263143898	0.481605736403381	0.775229010285204	0.209178053159811	36.8492043675496
TMIE	ENSBTAG00000052515	-7.14496785402895	-32.2320531948505	0.746704300909962	0.271334587578201	0.166251472053501	32.398304666904
NCCRP1	ENSBTAG00000014296	7.11259157979939	1.28225291262756	0.486041477498022	0.615406405211467	1.29406602372885	0.0118131111012905
OLFM4	ENSBTAG00000022779	6.98174767844643	13.837555827811	0.308109841450442	0.689536041445065	13.9525652777417	0.11500944993065
TDGF1	ENSBTAG00000021119	-6.80310364038778	-201.057849100285	0.310916583251406	0.631884126277704	1.65549732489853	202.713346425183
ENSBTAG00000011470	ENSBTAG00000011470	-6.77458040455884	-10.1650175378062	0.446871761062352	0.140478886939971	0.0729406735126094	10.2379582113188
PADI1	ENSBTAG00000002138	6.65159901658234	25.1419413304667	0.412782797072968	0.355129926047248	25.4312967007317	0.289355370265031
KRT13	ENSBTAG00000050581	6.60455651689265	48.8673317699611	0.47304553549609	0.518094262785813	49.4588326497406	0.591500879779518
SCG2	ENSBTAG00000021588	6.46281630849971	0.828607699462793	0.713710958282025	0.8	0.834794095323741	0.00618639586094831
LRP2	ENSBTAG00000004555	-6.4320396209309	-9.73441966584519	0.336120178665147	0.563394511740939	0.0974085128359711	9.83182817868116
ENSBTAG00000053748	ENSBTAG00000053748	6.30191233312157	53.7587482952646	0.556960222428914	0.39367890594909	54.5359006047368	0.777152309472178
KRT4	ENSBTAG00000012034	6.30006056405355	2.8479905970231	0.557329403416542	0.39367890594909	2.88921883625239	0.0412282392292932
GAL	ENSBTAG00000009393	6.25065974277691	332.298429497016	0.250359346249565	0.442329997859117	337.161000271159	4.86257077414229
SLC30A8	ENSBTAG00000052098	-6.20793360598155	-11.1683475480057	0.576497311585583	0.616842353894826	0.116853247037114	11.2852007950428
MGC138914	ENSBTAG00000014328	-6.20252448589179	-131.701416485042	0.511629942801625	0.290781077446007	1.53555954194665	133.236976026989
UPK2	ENSBTAG00000012750	6.17135102341042	0.509952115161376	0.590019242974515	0.635711877517133	0.51822726966561	0.00827515450423429

LOC522479	ENSBTAG00000050427	6.13045929224964	154.713158413611	0.434115962813147	0.319281627009887	156.990223838749	2.27706542513746
ENSBTAG00000055199	ENSBTAG00000055199	-6.11874699133316	-1.21966498637771	0.671609886916306	0.780052974541294	0.0130108581114812	1.23267584448919
IHH	ENSBTAG00000008452	-6.06063859427148	-6.41313863037376	0.50762878188206	0.790167344499239	0.0823521556816896	6.49549078605545
F10	ENSBTAG00000016385	-5.97778670464108	-1.82341483696047	0.722329456151623	0.631340237387627	0.0227455913127713	1.84616042827325
REG4	ENSBTAG00000032193	5.97254369580806	2.76426651309394	0.457878650885152	0.494784534101104	2.81112852797451	0.046862014880569
LOC112446672	ENSBTAG00000052798	5.97028153990243	0.422175917456848	0.568501932138137	NA	0.422175917456848	0
SPINK1	ENSBTAG00000015558	5.97021056305912	2.92933404999975	0.427337973985977	0.23488985350991	2.98042556807162	0.0510915180718691
SLURP1	ENSBTAG00000016209	5.96628107842237	2.13890518318835	0.462899263017627	0.468517291928679	2.17925251219645	0.0403473290080999
MSMB	ENSBTAG00000011660	5.9172246051464	11.559692840123	0.57438559357943	0.420535691388361	11.7680586379666	0.208365797843637
MMP3	ENSBTAG00000037768	5.90784242672446	0.300619796967037	0.575586118756744	0.687145987100115	0.305441167173454	0.0048213702064169
LOC101906048	ENSBTAG00000046283	-5.81406656406624	-112.22982739435	0.532964803569153	0.640889438458854	1.6756578031257	113.905485197476
TAAR1	ENSBTAG00000037718	5.69125738448153	5.30173948963706	0.350309420495675	0.338114887682562	5.41421185107781	0.112472361440749
BPIFA2C	ENSBTAG00000031376	5.66134798079537	6.93276385461759	0.439228909687748	0.198886695611993	7.08485654903631	0.152092694418717
EXD1	ENSBTAG00000008363	5.64090424776476	3.62173406094445	0.442225725513415	0.223980633222556	3.70258022965377	0.0808461687093241
KCNF1	ENSBTAG00000021280	-5.56880608077551	-0.733886478768648	0.588670714403363	0.3056205133675	0.0120916607764349	0.745978139545083
TMPRSS3	ENSBTAG00000048816	5.50926031435089	233.547919228969	0.296184512776969	0.523349310076629	238.835256077509	5.28733684853941
ENSBTAG00000048816	ENSBTAG00000020512	5.49427315123949	4.03539223846517	0.438178638298546	0.604198292230674	4.13164007802351	0.0962478395583349
GJB1	ENSBTAG00000008161	-5.46737867462709	-7.7516972071287	0.513730635930614	0.573761494647608	0.147320164154682	7.89901737128338
CLCA1	ENSBTAG00000052224	5.46220414840613	216.918610691352	0.348520323717683	0.220911275830118	222.367665187114	5.44905449576143
ENSBTAG00000052224	ENSBTAG00000046768	-5.44645341710979	-0.141385399020172	0.732679109278405	0.592853116464926	0.00158103991329466	0.142966438933466
IGFBP1	ENSBTAG00000031532	5.41407486190766	0.232438603880333	0.398708784245792	0.687781598208397	0.237886522437604	0.00544791855727039
ENSBTAG00000031532	ENSBTAG00000001392	-5.30610654888893	-9.21065752901083	0.392048787077624	0.729561457520693	0.198741578938214	9.40939910794904
RDH16	ENSBTAG00000014683	-5.30278893747946	-0.211260754246596	NA	0.22153142686123	0	0.211260754246596
APOBEC1	ENSBTAG00000038835	5.14904004851144	1.7300673491015	0.529195805451826	0.620588665099255	1.78304759578754	0.0529802466860465
LOC509961	ENSBTAG00000004588	5.125849712773	0.51405456520375	0.670915502609821	0.413939241131895	0.533021604361805	0.018967039158055
KCNN4	ENSBTAG00000022246	5.11845131628365	9.31715085297592	0.271822638609792	0.122277235259634	9.63955600286589	0.322405149889969
C29H11orf86	ENSBTAG00000018703	5.11490131443356	0.901634401094783	0.466235913936583	NA	0.901634401094783	0
OSTN	ENSBTAG00000039237	5.10472743361731	2.13381832461088	0.493917195234149	0.619924271681624	2.1905700177357	0.0567516931248251
ENSBTAG00000039237	ENSBTAG00000050026	-5.0899259170152	-0.466797520303263	0.857142857142857	0.748126166762856	0.00991140105104993	0.476708921354313
ENSBTAG00000050026	ENSBTAG00000047155	5.08257740147134	0.10570344761818	0.366920338844249	0.8	0.108275364342786	0.00257191672460578
C28H10orf71	ENSBTAG00000012621	5.05666166554248	0.623191000853263	0.430556596569112	0.608384191973237	0.643170830812637	0.0199798299593742
RTN4RL2	ENSBTAG00000039967	-5.02169556579284	-4.52817025221992	0.372114164867171	0.191777165832691	0.112258073916326	4.64042832613624
KRT78	ENSBTAG00000021240	5.01230629889222	5.32986970531806	0.543476717946431	0.33561895102292	5.5235494764874	0.193679771169345

DCSTAMP	ENSBTAG00000019636	-4.99771198991172	-1.75079853951409	0.595687858980708	0.370993004192074	0.0303553412939253	1.78115388080802
SCARA5	ENSBTAG00000039289	-4.99333979615527	-175.992664251122	0.34694681929318	0.376336987526026	5.40145840614963	181.394122657272
LOC527068	ENSBTAG00000053443	4.9914602241738	4.2131051169979	0.557702964942814	0.243677868243968	4.36668800060571	0.153582883607807
RUM1	ENSBTAG00000021280	4.98204833007017	9.74751598331487	0.150582097847591	0.262393338886956	10.0955034837624	0.347987500447485
IVL	ENSBTAG00000017827	4.98047792619673	3.53860191933584	0.56752775113844	0.635711877517133	3.6725988335046	0.133996914168757
ENSBTAG00000054600	ENSBTAG00000054600	4.97711136426043	10.2692488853657	0.326277886234182	0.345770984835542	10.6364735005654	0.367224615199633
CALML5	ENSBTAG00000013854	4.91059617065609	0.46970608591465	0.244710652791082	0.684360546693485	0.488532537211412	0.0188264512967618
LOC538679	ENSBTAG00000046482	4.90470651814782	0.397143436621251	0.263156523890617	0.615406405211467	0.415176626600994	0.0180331899797425
PENK	ENSBTAG00000004924	-4.82018704800901	-0.501637147293282	0.575389941722831	0.623033246461788	0.0142549202220615	0.515892067515344
TH	ENSBTAG00000026768	4.81970880567878	13.392416503268	0.761638079877615	0.190218988191608	14.0002827140149	0.607866210746869
A4GNT	ENSBTAG00000001451	4.80713439336761	0.300594879286845	0.435646804527321	0.258528707326984	0.314254997453264	0.0136601181664187
ENSBTAG00000048830	ENSBTAG00000048830	4.80228490133332	0.0270053431419432	0.423799663506785	NA	0.0270053431419432	0
BCAN	ENSBTAG00000015789	4.75558678325738	1.39685057289354	0.293854499177153	0.212551789148209	1.46213645276024	0.0652858798666992
CCL26	ENSBTAG00000052510	4.74406346202816	10.5467762364544	0.166608731000911	0.149745425037113	11.0033532317772	0.456576995322828
AGR2	ENSBTAG00000024406	4.74106289947718	4060.3690683971	0.188915372048269	0.211978516408433	4235.21089557	174.841827172901
ASPG	ENSBTAG00000017194	-4.68688328813046	-1.51479249813694	0.417312971349325	0.77426485970263	0.0486697215946323	1.56346221973158
RADIL	ENSBTAG00000021913	-4.67776436525556	-7.40588386577895	0.266722263387651	0.767587755940789	0.251688861121091	7.65757272690004
TMEM229A	ENSBTAG00000049382	4.66450793972288	0.192363626559942	0.441941247442293	NA	0.192363626559942	0
KRT6A	ENSBTAG00000039425	4.65937157668344	4.52392082311196	0.525127285425684	0.529879305124812	4.73351117031102	0.209590347199066
GJB5	ENSBTAG00000005717	-4.61265825636491	-5.08705466735846	0.514698079451125	0.700279354678321	0.186697989645475	5.27375265700394
ADM2	ENSBTAG00000054072	4.61120314060221	14.0931763721036	0.226673923658365	0.192053192614435	14.7995669229185	0.70639055081487
MCOLN3	ENSBTAG00000016982	-4.59926761289637	-1.53294796619728	0.614935535631122	0.439745674542315	0.0472139550344666	1.58016192123174
ENSBTAG00000005324	ENSBTAG00000005324	4.58816104654107	2.82899586567018	0.572272110442103	NA	2.82899586567018	0
IGFBP3	ENSBTAG00000003994	4.58517460230098	95.2488081081069	0.0994427776122484	0.294491073326218	99.7007290097979	4.45192090169103
DCT	ENSBTAG00000002300	4.56942007303733	0.148815689838838	0.781024732486731	0.8	0.15533952547402	0.00652383563518186
BSP3	ENSBTAG00000003886	-4.55507383233905	-0.640788200719888	NA	0.358662173213077	0	0.640788200719888
ENSBTAG00000048395	ENSBTAG00000048395	-4.55475871869254	-1.49175928917298	0.259681812333872	0.413284074808977	0.0679444069205873	1.55970369609357
CYP3A4	ENSBTAG00000052665	4.54947861024019	16.1959899325145	0.505185337093724	0.234091152098849	17.0043917722522	0.8084018397377
KLF17	ENSBTAG00000047871	-4.51816238250645	-1.18224713276154	0.679395546313255	0.232158498444094	0.0567615033820296	1.23900863614357
TNC	ENSBTAG00000000575	4.43688586273517	208.62727582452	0.377655272939868	0.387453477402939	219.345984954238	10.7187091297178
MGAT4C	ENSBTAG00000011153	-4.42830804103556	-10.1430414632334	0.444765811814567	0.393854200026938	0.457699191000382	10.6007406542338
KCNE1	ENSBTAG00000001150	4.41825885492678	0.283951444740196	0.667496459654903	0.8	0.29826234934035	0.0143109046001541
C28H10orf99	ENSBTAG00000050197	-4.40459288804529	-2.46284578703824	0.595524385461041	0.196760828426254	0.0925943051593968	2.55544009219764

SFN	ENSBTAG00000009223	4.38739338650954	14.219610237416	0.493299337159911	0.156621523347076	15.0037883414765	0.784178104060512
PRODH	ENSBTAG00000047676	-4.37823848956379	-8.38833718911427	0.165357870002746	0.24670042616056	0.360378147127657	8.74871533624193
S100A12	ENSBTAG00000012638	4.37468878868362	10.7508742455153	0.529950363454291	0.367741910941125	11.309453729421	0.558579483905632
ENSBTAG00000052585	ENSBTAG00000052585	-4.37085733781731	-4.65499638608366	0.529564529737433	0.566681904472133	0.172518301137926	4.82751468722159
SLC14A1	ENSBTAG00000019870	4.34841217170969	3.27389980244996	0.719294681521202	0.43878817341671	3.4405727956693	0.166672993219341
GJB4	ENSBTAG00000005719	-4.3460233148246	-9.2846910699956	0.455467368287012	0.441146566258235	0.390694171159466	9.67538524115507
ENSBTAG00000048794	ENSBTAG00000048794	4.34073589208589	167.677422864312	0.41365717422201	0.41744372950316	177.361731400924	9.68430853661206
LOC112441481	ENSBTAG00000012540	-4.33881125665703	-0.529486086435191	0.709098966057574	0.329646698327027	0.048095017148613	0.577581103583804
LOC507527	ENSBTAG00000013507	4.33049803539077	0.00796028178064769	0.562107108912761	NA	0.00796028178064769	0
ENSBTAG00000053707	ENSBTAG00000053707	4.3267015493717	1.58412444509021	0.40865370111719	0.444099991082275	1.67820105617526	0.0940766110850541
S100A8	ENSBTAG00000012640	4.31913547505644	1.19861552784099	0.5233191170937	0.38928140268617	1.26725393728352	0.0686384094425284

Table S4: The 100 most differently expressed genes in the follicular and luteal phase of the sheep cervix.

Gene	ID	**Log Fold Change**	**Dif Means**	gini_1	gini_2	Mean1	Mean2
ENSOARG00020011601	ENSOARG00020011601	7.65678771992481	2.33754749161547	0.767658467531659	0.735541205336112	2.35002171949169	0.0124742278762233
ENSOARG00020000537	ENSOARG00020000537	7.32707456273094	0.0753685444755734	0.81927013027439	0.736979654019151	0.0758769005296702	0.000508356054096724
KRTDAP	ENSG00000188508	7.25380690371756	0.0111377364888888	0.858832754411478	0.839786875344018	0.0111839536402679	4.62171513791126e-05
DRD2	ENSG00000149295	6.7507378119605	51.670365850602	0.463983997794401	0.853983800457149	52.1954422878483	0.52507643724623
SLURP1	ENSG00000126233	6.64203575141894	0.258738719182664	0.672064308043247	0.802490451645851	0.261554540361375	0.00281582117871167
KRT4	ENSG00000170477	6.64118502059344	1070.81788636707	0.825671836911915	0.924309206282594	1115.30013891217	44.482252545096
PLA2G4E	ENSG00000188089	6.57073948117742	0.594890875845066	0.870768628493842	0.875252767091832	0.596887198985571	0.00199632314050495
ENSOARG00020018856	ENSOARG00020018856	6.16812848914848	0.00738487708862217	0.892911726650831	0.697396395694228	0.00746780094259318	8.29238539710077e-05
MMP1	ENSG00000196611	6.16576596182193	0.403520062244577	0.676630866949708	0.894876015890183	0.40947511233817	0.00595505009359361
TECTB	ENSG00000119913	5.83895349961854	0.130225827049176	0.73503534592538	0.696393706901843	0.132679945325323	0.00245411827614712
SCG2	ENSG00000171951	5.65339614183538	0.867067709064849	0.540959058728606	0.831792221054691	0.885957372812962	0.0188896637481134
KRT79	ENSG00000185640	5.63144322349349	2.28717664250687	0.74623858964369	0.767969205304519	2.33717117174681	0.0499945292399396
CSN3	ENSG00000171209	5.62539017807886	1.12964207762822	0.899556216605685	0.916461904836629	1.13716401976393	0.0075219421357065
IBSP	ENSG00000029559	5.60558222079176	0.233812769438452	0.666056914977453	0.93875017133709	0.23617825645322	0.00236548701476805
ADAM7	ENSG00000069206	-5.58250234721926	-3.24039388002412	0.576242541355225	0.647289715558404	0.0654185677861933	3.30581244781032

ENSOARG00020019270	ENSOARG00020019270	5.56729164751594	0.146829870523219	0.955010580434334	0.817380378632078	0.147397625031332	0.000567754508112833
ENSOARG00020010220	ENSOARG00020010220	-5.50926952410315	-19.9830095240443	0.8423808079539	0.873602332007091	0.42262162265611	20.4056311467004
ENSOARG00020012069	ENSOARG00020012069	5.42856128341953	1.39833297284088	0.729774021844396	0.580709863778545	1.43393308416918	0.0356001113282972
ENSOARG00020024490	ENSOARG00020024490	5.42054576262215	0.201074255363184	0.85907376486467	0.834514981307652	0.206514545665752	0.00544029030256842
KRT85	ENSG00000135443	-5.36888720716065	-0.679769137156458	0.886335091400448	0.804029299987675	0.0144309064747076	0.694200043631165
TFF1	ENSG00000160182	5.31535865509055	2.77619608234766	0.673581585724974	0.709475018445018	2.85111580527535	0.0749197229276895
ENSOARG00020011654	ENSOARG00020011654	5.3040154599145	18.2264964452496	0.770570686141199	0.689710973149857	18.7332947083822	0.506798263132556
ENSOARG00020015596	ENSOARG00020015596	-5.195565115895	-1.29049973712559	0.887666111980304	0.84821302845308	0.0342772063486563	1.32477694347424
ENSOARG00020000114	ENSOARG00020000114	5.17939370895714	0.430830278181781	0.897266673501732	0.783973408492965	0.440758771530191	0.00992849334840928
ENSOARG00020000372	ENSOARG00020000372	5.10688287182257	0.0723453713139255	0.767810780167757	0.53851386930478	0.074657214528892	0.0023118432149665
ENSOARG00020024493	ENSOARG00020024493	-5.02464664754408	-1.01468859896477	0.416676548651069	0.669929118968995	0.030593915782405	1.04528251474717
ENSOARG00020013969	ENSOARG00020013969	-4.99117002843005	-0.0198389644560448	0.820235129618376	0.695395389932465	0.000601968203748093	0.0204409326597929
ENSOARG00020012116	ENSOARG00020012116	4.98002467282859	0.0279318533758113	0.857970026929464	0.942061535861	0.0282288172221723	0.000296963846360968
ENSOARG00020011773	ENSOARG00020011773	4.92106084845893	3.62959702073834	0.705832452761686	0.552839104353695	3.76124504961823	0.131648028879884
SLC26A3	ENSG00000091138	-4.89597054736947	-754.177499553705	0.330840174402812	0.57205899643128	24.6878518295235	778.865351383229
KLK4	ENSG00000167749	-4.78739708526638	-0.036818346797663	0.343745324886879	0.724051025161995	0.00130138372554361	0.0381197305232066
ENSOARG00020005727	ENSOARG00020005727	-4.7849627155198	-0.126265297680224	0.883704400412593	0.873124015145863	0.00447697990330126	0.130742277583525
ENSOARG00020002591	ENSOARG00020002591	4.76299285494152	0.466048532730646	0.678077825251925	0.764617399492265	0.486036335154241	0.0199878024235952
ENSOARG00020014156	ENSOARG00020014156	4.74941696212319	4.28441627026882	0.716880151074667	0.688276805760726	4.45875281379534	0.174336543526519
S100A8	ENSG00000143546	4.72531298633464	1.24619396428815	0.763615037471685	0.875275348415652	1.3678216532036	0.12162768891545
KRT36	ENSG00000126337	4.66482018841708	0.03647105778499	0.773783488764952	0.855531537929379	0.0376754035582404	0.00120434577325037
GRP	ENSG00000134443	4.66367170499564	35.9428408560887	0.836575551240455	0.924344533879189	41.3505939564768	5.40775310038808
ENSOARG00020021239	ENSOARG00020021239	-4.59940498848173	-2.25380931529806	0.931467139560216	0.843418264039823	0.0950856635040879	2.34889497880215
PRSS27	ENSG00000172382	4.54289695514717	4.2608055896385	0.920812849281677	0.717065701003756	4.32684874428226	0.0660431546437642
ENSOARG00020026387	ENSOARG00020026387	-4.48281311685366	-0.0633855437178787	0.607887537161391	0.774714330999619	0.00279937361951375	0.0661849173373925
TMPRSS11D	ENSG00000153802	4.47014188018446	0.320359227172443	0.851097623570003	0.759018701265936	0.340303618572474	0.0199443914000312
ENSOARG00020023153	ENSOARG00020023153	-4.46920291737012	-0.120826576423646	0.805968026338844	0.655413957691635	0.0053391274474405	0.126165703871087
RBP2	ENSG00000114113	4.41704619388933	1.30153487327226	0.558473998831547	0.40503014287533	1.37119757352733	0.0696627002550655
IGFL1	ENSG00000188293	4.35940626188571	0.019247284918464	0.69833725631857	0.816417173255253	0.0202812756483838	0.00103399072991973
CCK	ENSG00000187094	4.31328493529238	0.0117975985311752	0.518100524620428	0.701987852697961	0.0124758121098498	0.000678213578674543
GYS2	ENSG00000111713	4.3125787380265	0.369585906573251	0.524253710775918	0.685220095218447	0.390671053512615	0.0210851469393634
S100A12	ENSG00000163221	4.29146525792214	2.59106896214359	0.760076253812433	0.758523901877582	2.73791161414256	0.146842651998973
ENSOARG00020000383	ENSOARG00020000383	4.26760937076823	11.1569115886792	0.648259411010195	0.600321542206942	11.814051924527	0.657140335847787

ENSOARG00020011181	ENSOARG00020011181	-4.25900027561818	-0.00225588589752385	0.930692106722105	0.874560576071345	9.1217201147758e-05	0.00234710309867161
OXTR	ENSG00000180914	4.18480320699374	0.547423071976084	0.718277754306683	0.908907501438242	0.897956162912576	0.350533090936492
SPINK14	ENSG00000196800	-4.1817433953541	-0.00523798326827404	0.987012987012987	0.803486387697958	5.06417858976757e-05	0.00528862505417172
ENSOARG00020023117	ENSOARG00020023117	-4.17783768119593	-0.624688148962628	0.698154788809904	0.602559400142477	0.0342086856612063	0.658896834623835
TAC3	ENSG00000166863	4.16692906883266	4.6016944536264	0.321370647445723	0.77216717174452	4.89728885568738	0.295594402060981
ENSOARG00020025007	ENSOARG00020025007	-4.14445638002974	-8.14973070133269	0.572727687120471	0.58627115063701	0.45598327693389	8.60571397826658
LYPD2	ENSG00000197353	4.1303292704533	0.302506719475753	0.613558304566679	0.485074033372085	0.322127968800547	0.0196212493247939
ENSOARG00020020891	ENSOARG00020020891	4.08819605626998	92.8586085438018	0.462808121800076	0.484643228300231	99.1894542548288	6.33084571102699
KRT35	ENSG00000197079	4.05551448402891	0.758534796303049	0.401988086517413	0.628226461724914	0.809878475410767	0.0513436791077181
FSTL5	ENSG00000168843	4.03431199857308	0.608568192325194	0.503545104226829	0.742249302813963	0.649788221945426	0.0412200296202324
ENSOARG00020010586	ENSOARG00020010586	-4.02578428004074	-0.654413893237148	0.879163659198644	0.866364404754675	0.0415177637863426	0.69593165702349
MATN1	ENSG00000162510	4.01864242379543	0.0182033427716846	0.462937829355593	0.616734312399007	0.0194741891993515	0.00127084642766691
ENSOARG00020005568	ENSOARG00020005568	3.99996950903221	8.26166695100927	0.556216936306709	0.654621653406175	8.85229260051368	0.590625649504409
ENSOARG00020001244	ENSOARG00020001244	-3.9880188404984	-0.00787557057671479	0.795407438558841	0.777488005635453	0.000490330378307131	0.00836590095502192
KRT3	ENSG00000186442	3.95241187819827	0.11778235866841	0.836574943680045	0.712705203734278	0.126278816641448	0.00849645797303805
MMP12	ENSG00000262406	3.94563871231721	2.99494473103739	0.529945505351547	0.589261116546984	3.21901550161837	0.224070770580984
ENSOARG00020000427	ENSOARG00020000427	3.93442293948545	0.0762251651378047	0.768973657959708	0.887591724106844	0.0821005267021742	0.00587536156436946
ATP13A5	ENSG00000187527	-3.91530249608752	-226.767121964689	0.610019612038265	0.557756088370324	15.3212325177328	242.088354482421
KRT78	ENSG00000170423	3.84620142858859	4.85664628411337	0.68743710538597	0.510560392168535	5.24654141687767	0.389895132764298
TMIE	ENSG00000181585	-3.80542262850698	-0.501117361694343	0.594644543692849	0.520508056405506	0.0364539900621305	0.537571351756473
OLFM4	ENSG00000102837	3.79735004215817	4.118938668959	0.552708201468705	0.653505583247968	4.45856250784033	0.339623838881322
KRT75	ENSG00000170454	3.78583249517957	1.6396310134023	0.729285450242233	0.7241226686298	1.77754891603256	0.13791790263026
DKK1	ENSG00000107984	-3.78531830978448	-0.458407213984153	0.854845394778049	0.52261665445266	0.033951079305922	0.492358293290075
INHBE	ENSG00000139269	3.7554520487291	14.0326458083088	0.583151242725386	0.653289855793245	15.2311565181	1.19851070979119
IGFBP1	ENSG00000146678	3.75169586700212	0.0172064280114894	0.654167742730961	0.881112440505228	0.0183669947090168	0.00116056669752738
TTR	ENSG00000118271	3.73586741306458	0.0328280103927138	0.464159129645637	0.564666762616246	0.0355813833198303	0.0027533729271165
ENSOARG00020012854	ENSOARG00020012854	3.73388499719166	0.150547505319081	0.535012683751303	0.45452163732735	0.163738578529154	0.0131910732100728
BPIFA3	ENSG00000131059	-3.70338876419038	-0.0665372074337309	0.788751878193867	0.578375700922563	0.00518750493299057	0.0717247123667215
ENSOARG00020018060	ENSOARG00020018060	-3.61631363751709	-0.00234932725910245	0.96553961435552	0.514908196436354	0.000163382880576069	0.00251271013967852
NMB	ENSG00000197696	-3.6105884527357	-0.361948629550567	0.421000128689567	0.501549653653504	0.0302894106364112	0.392238040186978
ENSOARG00020005653	ENSOARG00020005653	-3.60931247910219	-0.00842281835341439	0.893464683857698	0.859496284174818	0.000577823043094457	0.00900064139650885
ENSOARG00020007834	ENSOARG00020007834	3.60820086544691	169.348643046171	0.917640829763144	0.570628887624536	174.100120125419	4.75147707924802
APOBEC3Z1	APOBEC3Z1	3.60065605987899	0.0818940483603901	0.941469453500167	0.843196888614979	0.0837095119055201	0.00181546354513008

AQP9	ENSG00000103569	-3.59738285500367	-86.7124577204827	0.696200949984435	0.43057366262928	7.32754508754471	94.0400028080275
NTS	ENSG00000133636	3.56316236884929	253.658027656422	0.50681913545105	0.797792179959098	278.697144954811	25.0391172983889
ENSOARG00020009811	ENSOARG00020009811	3.56249925596615	0.558084808281715	0.831713052585341	0.85159934279402	0.612167529601945	0.0540827213202299
GREM1	ENSG00000282046	3.55628746191299	3.35950016879503	0.647308286433341	0.525331956299854	3.70107558490097	0.341575416105935
ENSOARG00020012216	ENSOARG00020012216	-3.49032633160166	-18.1010250730951	0.601089691235662	0.554103952875788	1.68238105983719	19.7834061329323
SERPINB12	ENSG00000166634	3.47420778507579	0.192089898033723	0.556225483769819	0.56136095265624	0.212634844490066	0.0205449464563438
ENSOARG00020001753	ENSOARG00020001753	-3.43537988949491	-0.221251173376968	0.698080404451732	0.608363666918682	0.0210162606866806	0.242267434063648
ENSOARG00020018226	ENSOARG00020018226	-3.40717661733745	-0.0532386590988594	0.749107609830539	0.541064883342557	0.00522088185396742	0.0584595409528269
ENSOARG00020001098	ENSOARG00020001098	-3.40344001263939	-0.0342357893661368	0.830611709397833	0.730729588621107	0.00328916821087223	0.037524957577009
PHLDA2	ENSG00000274538	3.39106716508031	0.325306474319888	0.557379592939645	0.477502454620655	0.362979900512606	0.0376734261927181
ENSOARG00020007580	ENSOARG00020007580	3.38023399394846	14.6884611380242	0.916202274448986	0.547370017050209	15.1751004962149	0.486639358190693
ENSOARG00020008854	ENSOARG00020008854	-3.32043361197932	-14.3420626855636	0.613757392231996	0.504421717314006	1.51535317788897	15.8574158634526
UPK2	ENSG00000110375	3.31434756135508	0.00313727307173313	0.527111993891946	0.794120860470912	0.00352460930929406	0.000387336237560927
MMP7	ENSG00000137673	3.29494686122149	3.01760775813671	0.743589577048928	0.769719728775387	3.92029956663954	0.902691808502828
GRXCR1	ENSG00000215203	3.26416933728337	0.79133409904993	0.48311096262132	0.827128452731347	0.874049535012958	0.0827154359630285
GREB1	ENSG00000196208	3.26232532203146	976.398750184738	0.309089605914334	0.440305795982338	1099.41139428492	123.012644100181
ENSOARG00020008814	ENSOARG00020008814	-3.26060519749211	-0.244046050661095	0.755270394178281	0.548891648029976	0.0269150977481888	0.270961148409283
GJB6	ENSG00000121742	3.25789093422036	0.00483369541035877	0.733228680166457	0.827009795359644	0.00533650100566402	0.000502805595305254
ENSOARG00020000477	ENSOARG00020000477	3.24317205802819	0.0354886233817352	0.824143541036406	0.670801312676388	0.038286804915271	0.00279818153353582

Table S5: Genes exclusive to bovine cervix in the follicular and luteal phase

Gene	ID	**Log Fold Change**	**Dif Means**	gini_ 1	gini_ 2	Mean1	Mean2	estrus phase
BPIFA2B	ENSBTAG00000019752	8.01169488659356	0.627058853372568	0.495585367076419	NA	0.627058853372568	0	Follicular
LOC112446672	ENSBTAG00000052798	5.97028153990243	0.422175917456848	0.568501932138137	NA	0.422175917456848	0	Follicular
RDH16	ENSBTAG00000001392	-5.30278893747946	-0.211260754246596	NA	0.22153142686123	0	0.211260754246596	Luteal
C29H11orf86	ENSBTAG00000022246	5.11490131443356	0.901634401094783	0.466235913936583	NA	0.901634401094783	0	Follicular
ENSBTAG00000048830	ENSBTAG00000048830	4.80228490133332		0.423799663506785	NA	0.0270053431419432	0	Follicular
TMEM229A	ENSBTAG00000049382	4.66450793972288	0.192363626559942	0.441941247442293	NA	0.192363626559942	0	Follicular
ENSBTAG00000005324	ENSBTAG00000005324	4.58816104654107	2.82899586567018	0.572272110442103	NA	2.82899586567018	0	Follicular
BSP3	ENSBTAG00000003886	-4.55507383233905	-0.640788200719888	NA	0.358662173213077	0	0.640788200719888	Luteal
LOC507527	ENSBTAG00000013507	4.33049803539077	0.00796028178064769	0.562107108912761	NA	0.00796028178064769	0	Follicular
NPVF	ENSBTAG00000019447	4.19515510583535	0.0412841844376611	0.459296453953889	NA	0.0412841844376611	0	Follicular
ENSBTAG00000052976	ENSBTAG00000052976	-4.14806392999975	-0.135683077496103	NA	0.268062655301592	0	0.135683077496103	Luteal
ENSBTAG00000012533	ENSBTAG00000012533	4.12932092887349	0.00941592543141604	0.62449535675828	NA	0.00941592543141604	0	Follicular
ENSBTAG00000049959	ENSBTAG00000049959	3.72021199993998	0.00705352483514716	0.519300181975886	NA	0.00705352483514716	0	Follicular
LOC520402	ENSBTAG00000040320	-3.39489882153536	-0.0364767605915812	NA	0.393006256335909	0	0.0364767605915812	Luteal
KCNC2	ENSBTAG00000054123	3.36487699970871	0.0813137372751472	0.615161872409253	NA	0.0813137372751472	0	Follicular
MYL10	ENSBTAG00000026273	3.34370470861356	0.121832086532478	0.620855813182252	NA	0.121832086532478	0	Follicular
R3HDML	ENSBTAG00000013220	3.1956982497018	0.0211369823488593	0.614225023129965	NA	0.0211369823488593	0	Follicular
LOC523389	ENSBTAG00000053227	3.1156670054286	0.0553082185424062	0.440599361865036	NA	0.0553082185424062	0	Follicular
ENSBTAG00000040248	ENSBTAG00000040248	2.98069741015961	0.0126126783125132	0.422518946319597	NA	0.0126126783125132	0	Follicular
MC4R	ENSBTAG00000019676	2.77976167767441	0.181892877426001	0.647587296803692	NA	0.181892877426001	0	Follicular
ENSBTAG00000054106	ENSBTAG00000054106	-2.77866487828753	-0.032596894581462	NA	141339065763543	0	0.032596894581462	Luteal
ENSBTAG00000053691	ENSBTAG00000053691	2.67531653468796	0.00638802558021577	0.329641811491069	NA	0.00638802558021577	0	Follicular
ENSBTAG00000054917	ENSBTAG00000054917	-2.54230941311803	-0.0169661005586573	NA	0.23921970533918	0	0.0169661005586573	Luteal
